# DL-ADR: a novel deep learning model for classifying genomic variants into adverse drug reactions

**DOI:** 10.1186/s12920-016-0207-4

**Published:** 2016-08-10

**Authors:** Zhaohui Liang, Jimmy Xiangji Huang, Xing Zeng, Gang Zhang

**Affiliations:** 1 School of Information Technology, York University, Toronto, ON M3J1P3 Canada; 2 Second School of Clinic Medicine, Guangzhou University of Chinese Medicine, Guangzhou, 510120 China; 3 School of Automation, Guangdong University of Technology, Guangzhou, 510006 China

**Keywords:** Deep learning, Genomewide association study, Pharmacogenomics, Single nucleotide polymorphisms, Adverse drug reaction

## Abstract

**Background:**

Genomic variations are associated with the metabolism and the occurrence of adverse reactions of many therapeutic agents. The polymorphisms on over 2000 locations of cytochrome P450 enzymes (CYP) due to many factors such as ethnicity, mutations, and inheritance attribute to the diversity of response and side effects of various drugs. The associations of the single nucleotide polymorphisms (SNPs), the internal pharmacokinetic patterns and the vulnerability of specific adverse reactions become one of the research interests of pharmacogenomics. The conventional genomewide association studies (GWAS) mainly focuses on the relation of single or multiple SNPs to a specific risk factors which are a one-to-many relation. However, there are no robust methods to establish a many-to-many network which can combine the direct and indirect associations between multiple SNPs and a serial of events (e.g. adverse reactions, metabolic patterns, prognostic factors etc.). In this paper, we present a novel deep learning model based on generative stochastic networks and hidden Markov chain to classify the observed samples with SNPs on five loci of two genes (CYP2D6 and CYP1A2) respectively to the vulnerable population of 14 types of adverse reactions.

**Methods:**

A supervised deep learning model is proposed in this study. The revised generative stochastic networks (GSN) model with transited by the hidden Markov chain is used. The data of the training set are collected from clinical observation. The training set is composed of 83 observations of blood samples with the genotypes respectively on CYP2D6*2, *10, *14 and CYP1A2*1C, *1 F. The samples are genotyped by the polymerase chain reaction (PCR) method. A hidden Markov chain is used as the transition operator to simulate the probabilistic distribution. The model can perform learning at lower cost compared to the conventional maximal likelihood method because the transition distribution is conditional on the previous state of the hidden Markov chain. A least square loss (LASSO) algorithm and a k-Nearest Neighbors (kNN) algorithm are used as the baselines for comparison and to evaluate the performance of our proposed deep learning model.

**Results:**

There are 53 adverse reactions reported during the observation. They are assigned to 14 categories. In the comparison of classification accuracy, the deep learning model shows superiority over the LASSO and kNN model with a rate over 80 %. In the comparison of reliability, the deep learning model shows the best stability among the three models.

**Conclusions:**

Machine learning provides a new method to explore the complex associations among genomic variations and multiple events in pharmacogenomics studies. The new deep learning algorithm is capable of classifying various SNPs to the corresponding adverse reactions. We expect that as more genomic variations are added as features and more observations are made, the deep learning model can improve its performance and can act as a black-box but reliable verifier for other GWAS studies.

## Background

**Fig. 1:**
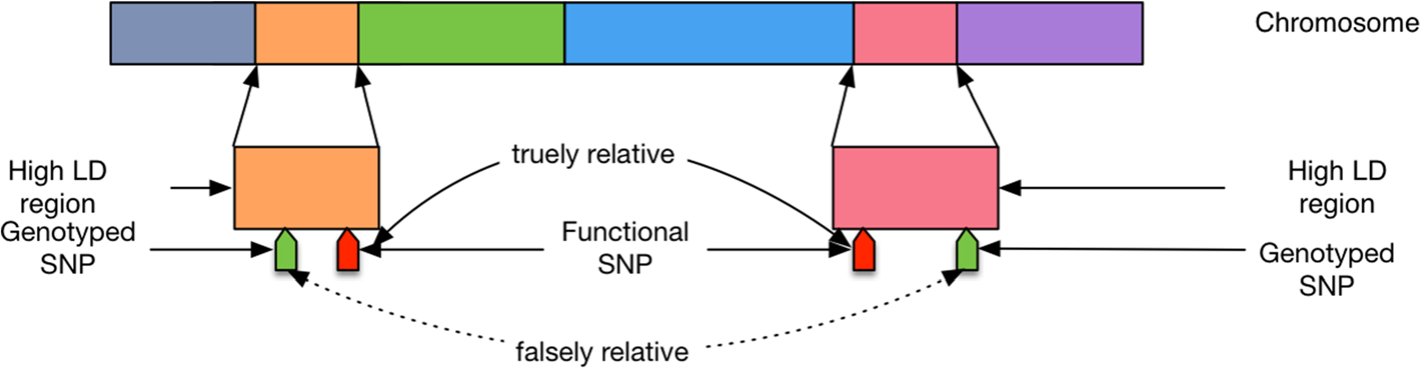
True and false association of SNPs

Genomewide association study (GWAS) is to explore the correlations among genomic variations and a series of genetic risk factors. It aims to reveal the complexity of the changes of DNA sequence and their corresponding effects on gene expression, proteins and finally leading to the macro factors such as disease susceptibility, prognostic factor and pattern of metabolism etc [1]. With the emergence of next generation of sequencing (NGS) and other improvements of genotyping and analytic technologies, the cost of genetic testing has decreased to reasonable cost-effective range for population-based GWAS study and personal genetic or whole genome testing [2]. The GWAS studies mainly deal with the complex associations between SNPs and attempt to measure and estimate the accumulative effect of relevant SNPs to biological systems. The SNPs can be markers to the changes of the macro systems or indirect factors that influence the system [1, 3]. Accordingly, the analytic strategies of GWAS can be categorized to inferential analysis and associative analysis. The typical inferential methods are the analysis of variance (ANOVA) and the Chi-square test (including Fisher’s exact test) which are to verify the associations between SNPs and the target events. And the latter methods include generalized linear model (GLM) approaches and multivariate logistic regression which are to select the more closely relative factors of SNPs to the target events from numerous candidates (usually in thousands of SNPs). However, the above methods are only designed to confirm the association between a specific SNP and a target event or a serial of related SNPs and a specific target event, which can be classified as a one-to-one or a one-to-many problem. They are incapable of solving the complex associative networks involving multi-dimensional correlations of dependents and independents, and those among independents and among dependents themselves. As shown in Fig. 1, the two chromosome segments (marked by orange and pink) can be associated with either the true association (the link between the red markers) or the false association (the link between the green marker). The false positive error will not be discovered because it also belongs to a high LD (Linkage Disequilibrium). These errors will accumulate as the associative network of SNPs and they will eventually generate error information that causes various problems. An example of this cost can be easily found in pharmacogenomics studies where the false linkage will cause either false prediction of risks or potential dangers of drug adverse reactions after the products are on the market.

The objective of pharmacogenomics is to study how the comprehensive genomewide variations (i.e. groups of relevant SNPs) systematically affect the patterns of pharmacokinetics and pharmacodynamics of individuals and the variation of biological patterns to the same or similar substances in different subjects [4]. It is widely believed that the SNPs on cytochrome P450 enzymes (CYP) are associated with individualized response and adverse reactions of many pharmaceutical and health products. For example, the combined variation of the CYP3A5 gene and breast cancer resistance protein (BCRP) can enhance the effect of rosuvastatin to decrease the blood LDL level and is hopeful to decrease the recurrence risk of cardiovascular disease [5]. Another study reported that the SNPs on CYP2B6 combined with ABCB1, SLC22A16 are associated with the toxicity and efficacy of doxorubicin and cyclophosphamide (AC) therapy for breast cancer [6]. The current studies summarize that the CYP polymorphisms on the specific loci CYP2A6, CYP2B6, CYP2C9, CYP2C19, and CYP2D6 are attributed to 20 % ~ 25 % the diversity of individual drug response. The associations have been extensively studied and characterized [7]. A research reported that the gene CYP2D6 affects 20 % ~ 25 % of the oxidative metabolism of clinical drugs [8]. In addition, SNPs affected by ethnicity can regulate the systematic biological functions of CYP. An epigenetic study in Mozambique found the distribution of the allele variants of CYP2B6 and CYP2C8 are homogeneous to other African populations, which implies some degree of homology [9]. An Asian study on the association of CYP and the interethnic variability of warfarin dosage revealed that the higher tolerance to warfarin of the Indian population can be explained by the combined influence of the SNPs of CYP2C9 and vitamin K epoxide oxidase reductase complex subunit 1 (VKORC1) [10]. A whole genome study on 96 Tibetans in China found the frequency of the CYP2D6*10 allele is lower than the other Chinese people belonging to the Han ethnic groups [11].

The literature review shows that with the disseminations of new genome sequencing technologies especially the deployment of NGS, the cost-effective of genome sequencing has reached a good ratio that makes large-scale individual studies feasible in both laboratory and clinical context. However, as a large sum of sequencing and SNPs data is generated at low cost, the conventional GWAS analytic methods have become the bottleneck for many study purposes that stress the complex association network connected to numerous SNPs and events with direct, indirect, unilateral and bilateral linkages. As indicated in Fig. 1, the available analytic methods are established to measure the one-to-one or one-to-many relations, but they are inadequate to measure the complex linkage in the multi-dimensional networks, which is the common purpose of pharmacogenomics studies.

**Fig. 2:**
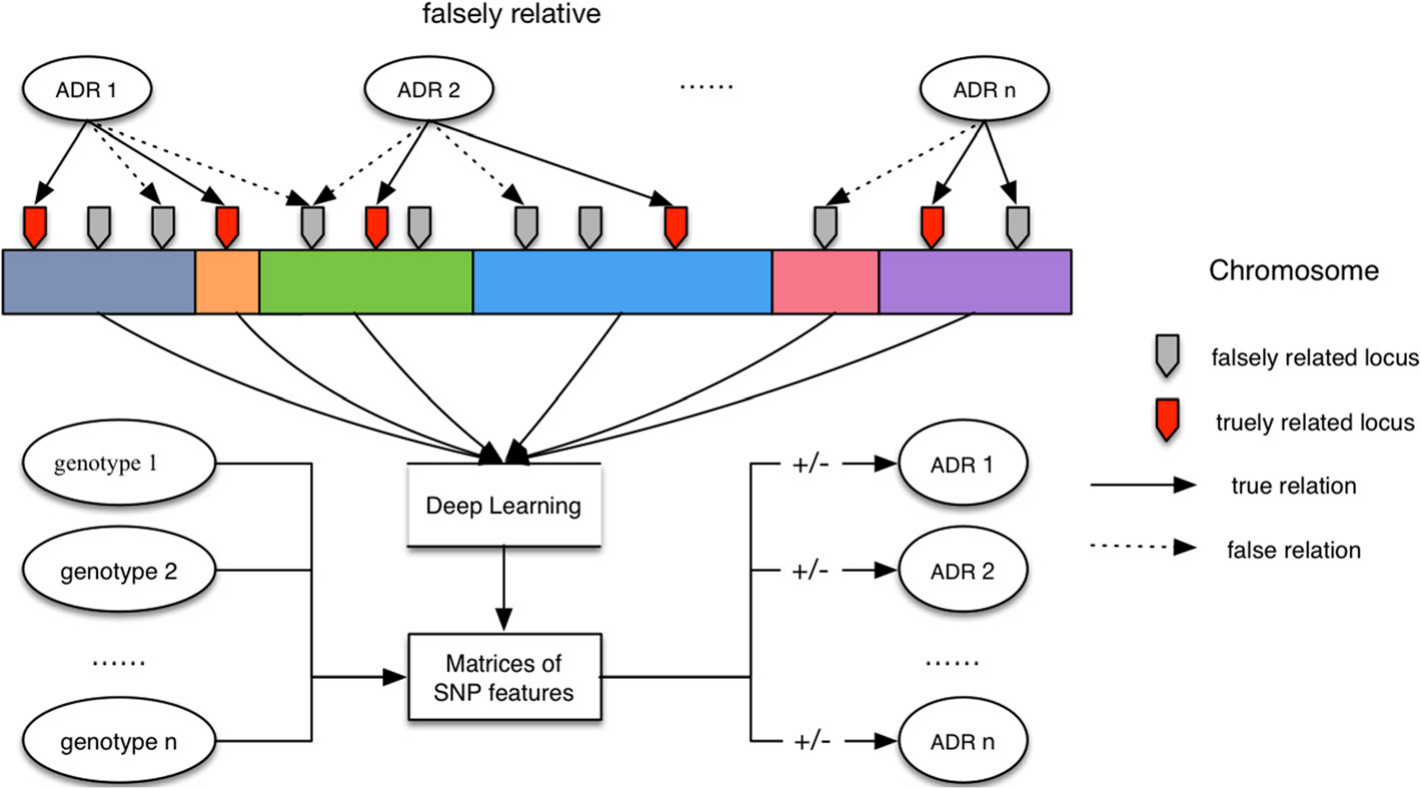
Classification of the associations network of SNPs and ADR

In order to solve this difficulty, we propose the machine learning method which can effectively seal the complexity of the SNPs and adverse drug associations into a computational model trained by empirical data. This study will analyze the complex associations among SNPs on two loci (CYP2D6 & CYP1A2) of cytochrome P450 enzymes and the occurrences of adverse drug reactions (ADRs) which are observed in a clinical observation. The goal is to demonstrate the proposed deep learning model can accurately classify the human participants with the different combinations of SNPs to the susceptibility of ADRs. The overall procedure is shown as Fig. 2, where both the true and false associations are put into the learning model. And we expect as more accurately labeled data are added to train the learning machine, the deep learning classifier will eventual render a reliable outcome with satisfactory accuracy.

## Methods

### Sequencing and ADRs

**Fig. 3:**
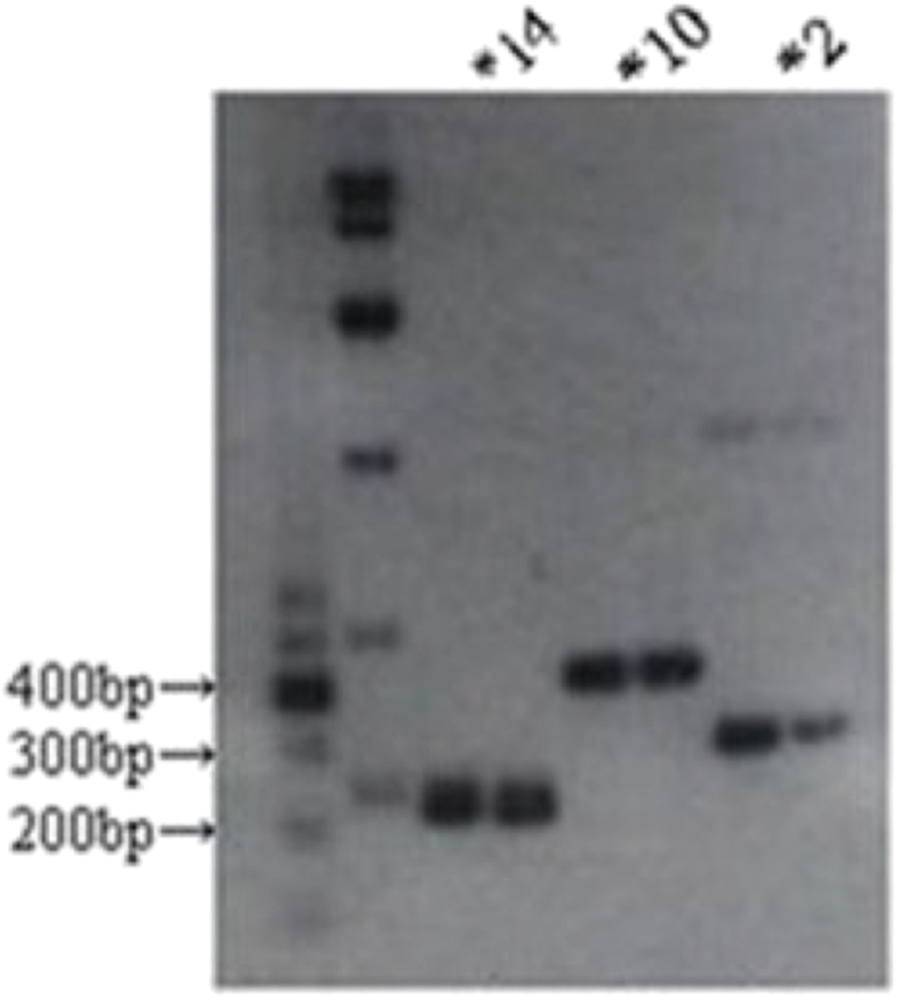
Gel of CYP2D6 alleles

**Fig. 4:**
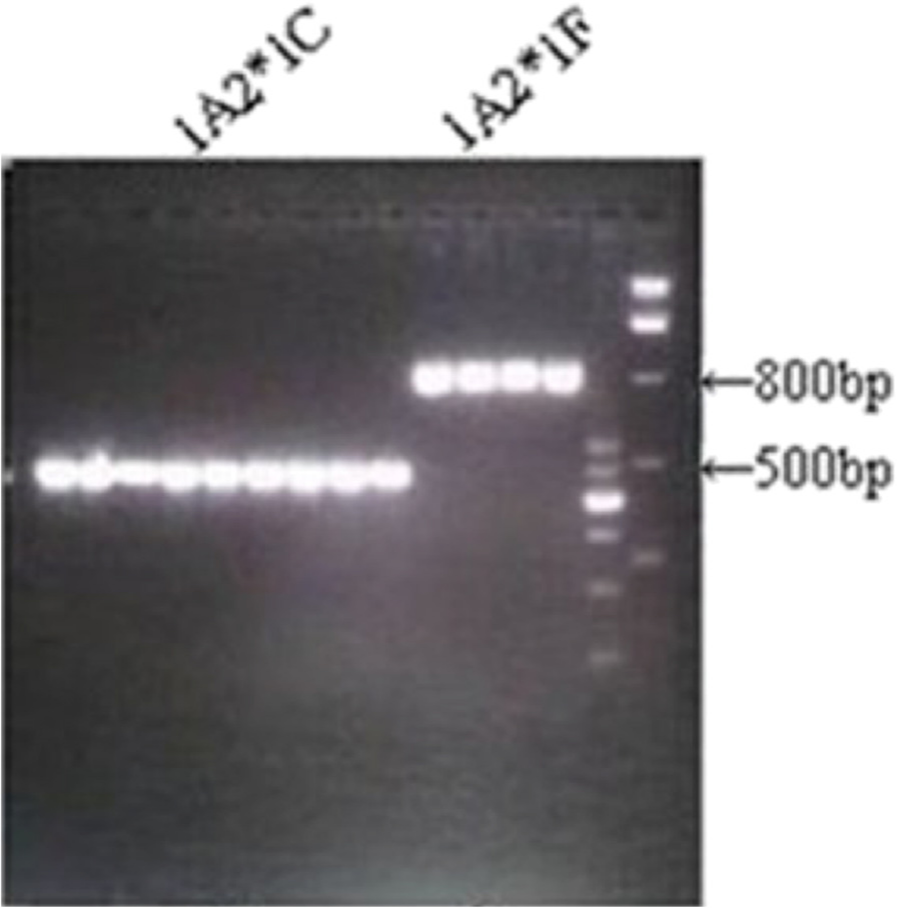
Gel of CYP1A2 alleles

The blood samples collected from the 83 human participants were kept in ultra-low temperature freezer before processing. The DNA was extracted and preserved at −80 °C. The alleles on the CYP1A2 and CYP2D6 gene were sequenced and genotyped by Polymerase chain reactions (PCR) with Pfu enzyme (using TianGEN Pfu PCR Mastermix kit). The sizes of the amplified five alleles of CYP2D6*2, *10, *14 and CYP1A2*1C, *1 F are respectively 312 bp, 443 bp, 235 bp, 597 bp, and 847 bp. The electrophoresis for the CYP2D6 locus was performed with 100 ng DNA in 2.5 % of agarose gel for 40 min. The gel with ladders for the CYP2D6 locus is illustrated in Fig. 3, where the alleles are respectively 235 bp (CYP2D6*14), 443 bp (CYP2D6*10) and 312 bp (CYP2D6*2). The electrophoresis for the CYP1A2 locus was performed with 100 ng DNA in 2 % of agarose gel for 60 min. The gel with ladders for the CYP1A2 locus is illustrated in Fig. 4, where the sizes of the alleles are respectively 597 bp (CYP1A2*1C) and 847 bp (CYP1A2*1 F).

The sequencing and genotyping of the two genes was done by Applied Biosystem 3130xl with the Invitrogen Bigdye® terminator v3.1 cycle sequencing kit (Life Technology). The frequency of different SNPs is presented in Table 1. It is noted that the CYP2D6*14 allele was not detected in this study due to the failure of genotyping in some loci in sequencing. Table 1 shows the sequencing results.

**Table 1:** Frequency of SNPs on CYP2D6 and CYP1A2

Genotype	Case Number	Percentage (%)
CYP2D6*2	74	100
CC	59	79.7
CT	9	12.1
TT	6	8.1
CYP2D6*10	83	100
CC	16	19.3
CT	8	9.6
TT	59	71.1
CYP1A2*1C	66	100
GG	38	59.6
GA	21	31.8
AA	7	10.6
CYP1A2*1 F	77	100
CC	33	42.9
CA	11	14.3
AA	33	42.9

There are 53 ADRs reported from clinical observation. The ADRs are categorized into 14 groups as listed in Table 2.

**Table 2:** Report of ADRs

ADR category	Number of Case (%)
Abnormal platelet counting	3 (5.7)
Abnormal protein counting	8 (15.1)
Abnormal TBIL	4 (7.5)
Abnormal neutrophil ratio	6 (11.3)
Abnormal lymphocyte ratio	7 (13.2)
Fecal occult blood	5 (9.4)
Abnormal fibrinogen	4 (7.5)
Prolonged PT	6 (11.3)
Abnormal blood chlorine	3 (5.7)
Abnormal hemoglobin	2 (3.8)
Abnormal RBC	2 (3.8)
Abnormal urobilinogen	1 (1.9)
Urine protein	1 (1.9)
Abnormal APTT	1 (1.9)
Total	53 (100)

### Modeling and data preprocessing

In order to explore the association of ADRs and SNPs on the two target, two data sets are set up respectively for data conversion of the trial group and the blank group. The data include: group ID, doses, the genotypes of the alleles and all reported ADRs. The five alleles (CYP2D6*2, CYP2D6*10, CYP2D6*14 CYP1A2*1C and CYP1A2*1 F) of the two loci are coded by 15 dummy variables to indicate specific allele combinations of the of the diploid (i.e. wild type, homozygous and heterozygous), where we use “1” to represent a positive result to the corresponding allele and use “0” to represent a negative result to the corresponding allele. Accordingly, we use ordinal variables to represent the ADRs, where a “2” means an ADR with extremely increased level, a “1” means ADR occurrence with increased level, a “0” means no ADR occurrence, a “−1” means an ADR with decreased level, and a “−2” means ADR with extremely decreased level. All missing data are filled with “0” too. This preprocessing strategy will not add extra information to the model and thus it minimizes the influence to the outcome of data analysis.

### Generative stochastic networks

**Fig. 5:**
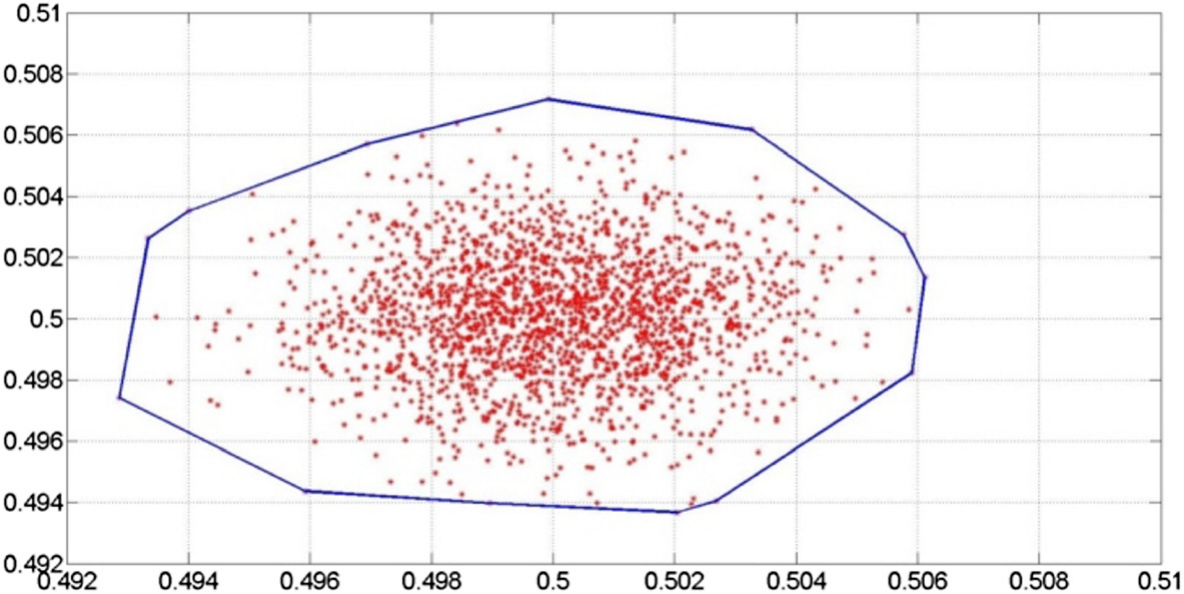
Two dimension Gaussian distribution

In the generative stochastic networks (GSN) *P*(*X*), we assume *X* = (*J*, *G*, *R*), then *P*(*X*) can be modeled by a given training of observed samples. Since the training data set *D* is acquired from different individuals, we can assume them as independent from *P(X)*. In order to model *P*(*X*|*D*)*,* we use a Markov chain formed by the data points. The transition matrix between the points is considered reflecting the ground truth distribution of *P(X)*. A two-dimension Gaussian distribution that contains 2000 states points is illustrated in Fig. 5.

Then we assume *X*^−^ is a sample set independently from *P*(*X*). The probability of *P*(*X*|*X*^−^) is calculated by the Bayes' theorem. It is noted that both *P*(*X*|*X*^−^) and *P*(*X*) can be re-evaluated and be used to produce another distribution of sample space if the prior distribution *P*_0_(*X*|*X*^−^) is known. Their relation is presented by $P\left(X\Big|{X}^{-}\right)=\frac{1}{\beta }{P}_0\left(X\Big|{X}^{-}\right)$ where *β* is a constant independent of X.

Based on the above assumption, we can apply the GSN model with a denoising auto-encoder (DAE) with the parameters *x*_*i*_ ~ *P*_0_(*X*^−^|*x*_*i*_) and *x*_*i* + 1_ ~ *P*(*X*|*x*_0_, *θ*), where *x*_*i*_ is the *ith* outcome regarding the probabilities of ADRs of the study, and *θ* stands for the parameters (mean and covariance) of the Gaussian distribution. A hidden variable *H*_i_ is assumed to govern the result *x*_*i*_ via a serial of unknown associations. Thus, the hidden Markov chain with X and H as its state variables can be expressed by Eq. (1) and Eq. (2):

1 $$ {H}_{i+1}\sim P\left(H\Big|{H}_i\right),{x}_i,{\theta}_1 $$

2 $$ {x}_{i+1}\sim P\left(X\Big|{H}_{i+1},{\theta}_2\right) $$

In order to launch the hidden Markov chain, we need the initial values of the hidden variable *H*_*0*_ and which can determine x0 and H1. Unfortunately, the value of H0 is not directly acquired because we do not have direct information regarding H0. An alternative method to get the value of *H*_*0*_ is to set it as a constant [14], but in this study, we assume that H0 is determined by the prior knowledge via some methods. Assume x* to be the mean of all x_i_ ∈ X and M* to be the co-variance of X, then the mode of H0 can be calculated with x* and M* and consequently the Gaussian distribution can be determined by H0. Based on the idea of Bengio et al., the following theorem can plot the main property of the GSN model defined by Eq. (1) and Eq. (2) [15].

Given (*H*_*i*_, *x*_*i*_)_*i* = 0_^*N*^ is a hidden Markov chain defined by Eq. (1) and Eq. (2). If *N* is big enough, we can define a stationary distribution ∏(*H*, *x*) where the samples of X determined by (*H*, *x*) comes from the same distribution *X*_*0*_. Thus we can build the connection between the GSN model and a deep network to generate data samples based on the distribution of the original set. A denoising auto-encoder (DAE) is set up to train the model, and to sample and evaluate its consistency. The purpose of training DAE is to predict *X* under the given distribution *P*(*X*|*X*^−^, *θ*_*i*_) where X^−^ is from a sample set. *P* is a distribution affected by *θ*_1_, which can be a normal distribution or a *t*-distribution. The training of DAE is a general Bayesian procedure with a maximal likelihood regularization term. Eq. (3) represents the expected value of the joint distribution of X:

3 $$ P\left(X,{X}^{-}\Big|{\theta}_1\right)=P(X){P}_0\left({X}^{-}\Big|X,{\theta}_1\right) $$

Then according to the Gibbs sampling theory, the procedure of sampling is presented as:

4 $$ {x}_i\sim P\left(X\Big|{x}_{i-1},{\theta}_1\right) $$

5 $$ {x}_i^{-}\sim {P}_0\left({x}^{-}\Big|{x}_i\right) $$

where *x*−is a data sample acquired from *P*_*0*_*(X)*. Let *Tj* be the transitional operator of the hidden Markov chain:

6 $$ {T}_j\left({x}_i\Big|{x}_{i-1}\right)={\displaystyle \int }{P}_{\theta j}\left({x}_i\Big|{x}^{-}\right){P}_0\left({x}^{-}\Big|{x}_{j-1}\right)d{x}^{-} $$

And let T* be the ground truth transitional operator of the hidden Markov train where

7 $$ \left|\left|\left({T}^{*}-{T}_j\right)\alpha \right|\right|{}_2\le {\left|\left|{T}^{*}-{T}_j\right|\right|}_2\to 0 $$

In the end, we get *P*_*θn*_(*X*|*X*^−^) → *P*(*X*|*X*^−^) when *n* → ∞. In addition, *α* is a control parameter determined by dependent on DAE. Eventually, we can implement the above steps by Algorithm 1.

As shown above, the training subsets are generated by a Gibbs sampling procedure in order to measure *P* (Line 3 to 7). And it is a generative distribution. During this procedure, the original training Set *D* is extracted from the sample set simultaneously (Line 8 to 10). *P* is a Gaussian distribution tuned by the parameter *θ*. The algorithm will render the *θ* value and a stacked DAE of *r* layers. The algorithm scans the training set *D* rendered by the code in Line 10 to 18 in order to tune the stacked DAE to keep it consistent with *P*. The training of DAE applies a stepwise algorithm, and we can adopt the strategy to train the stacked DAE layer by layer, therefore, the encodings can be restored to original inputs as much as possible through the trained DAE [15]. Through the number of layers of stacked DAE is defined as *r*, the numbers of inputs and outputs are not determined by parameters. Random numbers are in the range of [2*d*, 5*d*] where *d* stands for the dimension of training data sample. Finally, the time complexity of the algorithm is a polynomial function of *D* set at the beginning.

## Results

In order to evaluate the effectiveness of the new algorithm, we provide two conventional algorithms from the previous studies [12, 16] for comparison. The first one is a baseline method based on a least square loss (LASSO) algorithm to establish the connection between the SNPs and ADRs [12]. This method adopts a special minimum least square loss procedure with a hinge loss constraint to identify the model parameters. It assumes there is some polynomial relationship between the observed SNPs and ADRs, and thus the LASSO algorithm is able to get a parameterized discriminate function between the inputs and the outputs. The same data set was used to test and evaluate the LASSO algorithm as in this study. The second comparison algorithm is k-Nearest Neighbors (k-NN) implemented by Li et. al [13]. In their study, k-NN is used to solved the problem by treating it as a multiple target regression. The k-NN model applied directly predictions on a sample set based on the whole the training set, where k-NN does not determine a function to solve the problem but instead it implements a transductive learning procedure. In our evaluation, the LASSO algorithm is labeled as M, the k-NN algorithm is labeled as M2, and the proposed GSN generative algorithm is labeled as M3. It is noted that the results generated by these algorithms are the distributions of probabilities.

There are two evaluation criteria. The first one is the prediction accuracy which is to assess the overall performance of the algorithm. The second one is the impact on the model performance of noise to the predictions associated with the sample size of the training set. In order to indicate the predictive accuracy of the three model, we need to evaluate the loss of accuracy through the predictions where the loss of accuracy is the difference between the ground truth and the predictive value. The accumulative effect of the loss of accuracy through a single experiment with *n* test samples can be indicated be the average of accuracy loss defined in Eq. (8):

8 $$ {l}_a=\frac{1}{n}{\displaystyle {\sum}_{i=1}^n}\left|\frac{h\left({x}_i\right)-{y}_i^{*}}{y_i^{*}}\right. $$

Where ***y***_i_^*^ stands for the ground truth value related to ***x***_***i***_, and the value of ***l***_***a***_ is the average of the *n* tests.

Table 3 presents the results of the accuracy predictions by the three algorithms. In order to present the results consistently, we list the average accuracy losses in each test, where a higher ***l***_***a***_ reflects less accurate of the corresponding prediction.

**Table 3:** Prediction Accuracy of the 3 Models in *l*_*a*_ (%)

ADR category	M1	M2	M3
Abnormal platelet counting	16.2	18.6	13.9
Abnormal protein counting	18.4	15.2	15.0
Abnormal TBIL	16.9	14.8	14.5
Abnormal neutrophil ratio	13.2	13,7	11.0
Abnormal lymphocyte ratio	12.9	14.3	11.4
Fecal occult blood	17.8	18.9	16.1
Abnormal fibrinogen	14.7	15.0	12.9
Prolonged PT	15.9	18.9	14.6
Abnormal blood chlorine	14.7	16.0	14.1
Abnormal hemoglobin	20.6	20.9	18.7
Abnormal RBC	15.7	14.9	13.4
Abnormal urobilinogen	21.8	19.9	17.5
Urine protein	20.1	21.6	19.8
Abnormal APTT	14.6	13.7	12.5

**Fig. 6:**
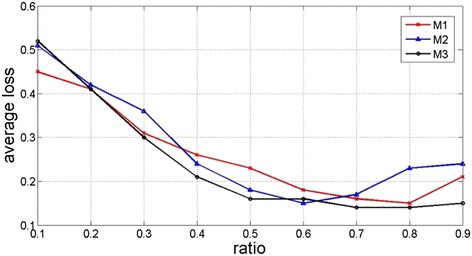
Comparison of average loss of Accuracy in different training/testing set ratios

The results from Table 3 indicate that in the prediction of each category of ADR, the new GSN generative algorithm (M3) has the best performance for it has the less average accuracy losses (*la*) in the predictions to all categories compared to M1 (LASSO algorithm) and M2 (k-NN algorithm). In the evaluation of the impacts of sample size and noise, we continuously change the volume of the training set and noise, and then we compute the average accuracy losses (*la*) given a specific ratio of the training set and test data set. The whole data set is respectively partitioned into the training set and test data set with the ratios from 1:9, then 2:8, and to 9:1, where the data points are randomly selected. The test results are shown in Fig. 6. The data indicates the prediction accuracy of all three models increase (as the value of *la* decreases) when more data are allocated to the training set. The GNS generative algorithm (M3) has the lowest average loss (*la*) when the training/testing set ratio is over 0.2. This indicates that M3 starts and keeps having the best performance over the three algorithms when the training set is 20 % in size of the testing set.

**Fig. 7:**
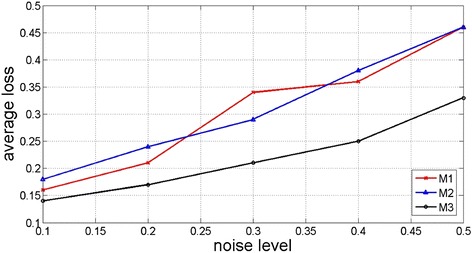
Comparison of the average loss at different noise levels

To evaluate the influence of noise level to model performance, we divide the whole data set evenly in half for training and testing (i.e. the training/testing set ratio is 5:5). The Gaussian noise is assumed to affect all the features of the sample points in the training sets. Since the stratification effects brought by the factors such as ethnicity, geographic region, and social environment can be adjusted by expanding the whole data set, and no systematic bias is identified, we assume all the features are independent and thus the noise can be effectively controlled by the mean and variance estimated based on Gaussian distribution. Let *a* be the average value of a given feature. The algorithm changes the mean from 0 to 0.5a with the step of 0.1*a*. And the variance is defined as half of the corresponding mean. By the above settings, the impact of noise on the prediction can be evaluated by observing the average loss of accuracy (*l*_*a*_) at different noise levels. The evaluation results are illustrated in Fig. 7.

The data from Fig. 7 indicates that the new GSN generative model (M3) has less impact on the noise since it has the lowest accuracy loss through all noise levels. This can be explained by the nature of the probability models which makes it less sensitive to noise compared to the LASSO algorithm which uses a discriminate function and the k-NN algorithm which uses a transductive function. Therefore, we conclude the new GSN generative model has the best performance and is capable of minimizing the effects of Gaussian noise.

## Conclusions and discussions

Genomewide association study or GWAS is one of the main trends in genomics research. GWAS aims to explore the variations across the human genome in order to identify the genetic risk factors associating with specific events in health (e.g. disease, ADRs, metabolic patterns, etc.) and to generalize the research results in the population. GWAS provides a valuable solution for pharmacogenetics whose goal is to identify the DNA sequence variations or SNPs and their association with drug metabolism, efficacy and adverse effects [1]. The conventional analytic strategy of GWAS focuses on exploring the relation between a single or multiple SNPs to a specific risk factor, which can be confined as the one-to-many. These studies consequently apply the inferential statistical models such as ANOVA (for quantitative) and Chi-square test (including Fisher’s exact test, for qualitative data) to verify these relations or use generalized linear model (GLM) or multivariate logistic regression to select the factors with statistical significance. However, the associations linking the genomic variants to efficacy and adverse effects are neither linear nor directly related. As indicated in Fig. 2, some identified associations between a SNP and an ADR are false but they have significance in the statistical tests since they are located in the same high LD (linkage disequilibrium) region. In addition, the SNPs that affect the same ADR might have mutual internal associations, and furthermore, the links and associations among the SNPs in a group or between different groups of SNPs are too complex to be explained by any single associative models because of the complexity of the internal many-to-many relations. This complex association network with SNPs and ADRs is most likely to provide numerous information to interpret or predict the individual response of the people with a certain genomic patterns (i.e. showing similarity in their genome to a certain degree) to a specific product. Thus, it is among the main interests of both health researchers and the pharmaceutical industry.

One solution to reveal the associations inside the network of SNPs and ADRs is to use machine learning methods in which the complex internal relations can be concealed into a black box. After trained by the labeled data set, the classifier will develop a capacity to differentiate the latent patterns and label the new data. In this study, we propose a deep learning model based on Generative Stochastic Network (GSN) as the implementation to solve the associations between the SNPs on two loci of cytochrome P450 enzyme and the ADRs in clinical observation. The generative model is considered more cost-effective compared to the conventional Bayesian models because it does not need to compute the joint likelihood and posterior distribution at a high computational cost. The GSN will learn the transition operator of a hidden Markov chain via the labeled training set, and the probability distribution of the training set can be estimated by the stationary distribution of the transition operator learned from the training set. This generative model is more efficient compared to the Bayesian algorithms because in a certain state of the Markov chain the transition operator is conditional on its previous state thus it only needs to compute a small step between the current and former states with a significantly lower cost at computation compared to the Bayesian models.

On the other hand, GWAS requires a large sample of observations covering numerous SNPs (i.e. thousands of genetic loci for example) in order to acquire a result of associations between SNPs and the due events with an acceptable degree of power. This usually causes the potential problem of a false positive result because except for the high cost of organizing a large-scale clinical study, the SNPs truly related to the risk factor are likely to be confounded by the false relations of the SNPs in the same or the adjacent high DL region. This risk is unable to be effectively prevented by the current mainstream GWAS analytic methods so far. The advantage of using machine learning model to classify the associated and unassociated SNPs is that the model performance is expected to be enhanced by increasing the size of the training set which can be acquired from empirical data. The GSN based deep learning model shows its robustness in that it is insensitive to system noise compared with other non-generative models. And this feature is important to classify a skewed sample.

The current studies imply that the cytochrome P450 enzymes play an important role in the metabolism of most drugs [4–11] and the SNPs in the loci are CYP2A6, CYP2B6, CYP2C9, CYP2C19, CYP2D6, CYP1A2 and CYP3A4 [7]. Though this study only selects two loci on CYP1A2 and CYP2D6 due to the limitation of the sequencing technology used for genotyping (i.e. PCR), the evaluation results reflect that the performance of GSN generative model will remain reliable and robust if more features are added. Additionally, the deep learning model will demonstrate its merits in large scale computing if bigger data sets are added to the model.

The uncertainty of ADRs is one of the major threats to healthcare. The economic loss caused by various ADRs relevant to medications is over 100 billion dollars annually in the US, and the expense of treatments for ADRs are actually comparable to the cost of the normal healthcare [17, 18]. A systematic review in the US reported that 86 % for the ADRs are related to the SNPs of cytochrome P450 enzymes [19]. Many current studies believed that the SNPs of cytochrome P450 enzymes are associated with the risk of ADRs and further related to the susceptive population. If a reliable strategy can be found to analyze the complexity of these genomic patterns and to render a predictive risk level to a serial of ADRs, it will hopeful lower the risk of ADRs both in new product development and clinical medication.

The study results indicate the GSN based generative algorithm is able to provide reliable and accurate predictions of risk levels to different ADRs after the deep learning model is trained by a relatively small data set [20]. As implied by our previous experiment [20], the deep learning model in this study shows its superiority in noise resistance and reliability over the convention models which requires the analyzed data sets in specific distributions or with low noise information [21–23]. Therefore, we believed the deep learning algorithm will provide an effective solution for the data complexity of GWAS in the short future.
